# Special Issue Editorial: “Antibacterial Agents from Natural Sources”

**DOI:** 10.3390/molecules29030644

**Published:** 2024-01-30

**Authors:** Carlos Henrique G. Martins

**Affiliations:** Laboratory of Antimicrobial Testing, Department of Microbiology, Institute of Biomedical Sciences, Federal University of Uberlândia, Uberlândia 38405-320, MG, Brazil; carlos.martins2@ufu.br; Tel.: +55-34-32391363

Antibacterials derived from natural sources represent a fascinating and promising field of research in the quest for effective and sustainable solutions to bacterial infections. Nature has long been a source of compounds with antibacterial properties, providing a rich reservoir for the development of new therapeutic agents. Plants, microorganisms, marine organisms and other natural products have been a traditional source of antibacterial compounds, producing bioactive substances that exhibit antibacterial activity. These natural agents not only combat bacterial growth but also potentially reduce toxicity and produce fewer side effects compared to synthetic alternatives.

As researchers delve into the molecular and chemical complexity of natural antibacterial agents, there is hope of discovering new therapeutic options. The synergy between traditional knowledge of natural products and modern scientific methodologies has the potential to provide innovative treatments for infectious diseases, bridging the ancient wisdom of nature with cutting-edge advances in contemporary medicine.

Standard therapies often have side effects, and are ineffective against strains resistant to existing antibiotics. Consequently, it is imperative for the microbiological community to develop novel therapies to combat these pathogens. Natural products have attracted research interest for decades due to their extracts and isolated molecules presenting biological properties of scientific interest [1] making them instrumental in the quest for antimicrobial agents capable of exerting safe and effective actions. Introducing scientific innovations regarding bioactive compounds with antimicrobial properties, the Special Issue “Antibacterial Agents from Natural Sources” comprises twelve unprecedented scientific articles, contributing to the advancement of scientific knowledge in this field. In this editorial, we address the main theme of each of the articles in chronological order of publication.

The first article in this Special Issue is by Nguyen et al., who assessed the antibacterial potential of *Persea Americana* (Maluma avocado) against Gram-positive and Gram-negative bacteria and demonstrated the antioxidant activity of Maluma. In the second article, Zhang et al. evaluated the antibacterial and antibiofilm activity of extracts from the leaves of three products: *Aphanamixis polystachya*, *Toona ciliata* and *Melia azedarach*, against Methicillin-resistant *Staphylococcus aureus* (MRSA) strains. Promising results were obtained for the *A. polystachya* and *M. azedarach* extracts, with *A. polystachya* being the most active against MRSA. The next article, written by Chan and Chong, also focused on MRSA. This time, the study utilized extracts and isolated fractions from the fruiting bodies of *Ganoderma boninense*, the mushroom, which, in turn, exhibited strong antibacterial activity in the assays conducted by the authors.

The sesquiterpene lactones present in the roots of *Inula helenium* L. were evaluated by Kenny et al. and were found to exhibit antibacterial activity against *S. aureus*. Meanwhile, in the next contribution, *Boswellia* sp. was studied by Jaros et al. The authors utilized boswellic acids present in the plants to assess antibiofilm activity against strains of *S. epidermidis*, *Enterococcus faecalis* and *Escherichia coli*, also demonstrating an additive effect in the combination of boswellic acids with antibiotics in combating bacterial biofilm. The activity of the extract from *Gnaphalium hypoleucum* DC, used in culinary dishes as a vegetable, and its major compounds in suppressing the quorum sensing activity of *Chromobacterium violaceum* was evaluated by Li et al. (contribution 6). This resulted in the selection of two compounds, apigenin and luteolin, which demonstrated the ability to inhibit the quorum sensing of the bacteria.

In their study, Halabi et al. evaluated the antioxidant and antimicrobial properties of *Phoenix dactylifera*. The authors conducted assays using bacteria and fungi, including *Pseudomonas aeruginosa*, *Acinetobacter baumannii*, *Proteus vulgaris*, *S. aureus*, *Citrobacter freundii*, *E. coli*, *Enterobacter aerogenes*, *Candida albicans* and *Klebsiella pneumoniae*. The fruit extract was considered as a potential bioactive compound. Santiago et al. (contribution 8) evaluated the extract of Brazilian red propolis against the bacteria *Helicobacter pylori*. Through in vitro and in vivo assays, the authors determined that the extract exhibited promising action against the bacteria, influencing the immune response of infected rats. In their contribution, Audah et al. evaluated extracts from the leaves of *Sonneratia caseolaris*, *Avicennia marina*, *Rhizophora mucronata* and *Rhizophora apiculata* against MRSA, in addition to assessing antioxidant activity. The ethanol extract from the leaves of *S. caseolaris* showed the highest antioxidant and antibacterial activity against MRSA.

An antiseptic produced from natural sources, xylityl sesquicaprylate, was used by Nogueira et al. in an alcohol-free mouthwash formulation to determine its antimicrobial action against the bacteria *Actinomyces viscosus*, *C. albicans*, *Fusobacterium nucleatum*, *K. aerogenes*, *Porphyromonas gingivalis*, *Prevotella intermedia*, *Streptococcus mutans* and *Tannerella forsythia*. The authors considered the efficacy profile of the mouthwash interesting, particularly regarding its efficacy against *A. viscosus*, *F. nucleatum*, *P. gingivalis* and *T. forsythia*. In their work, Al-Khayri et al. performed in silico evaluation of eighteen bioactive molecules from *Andrographis paniculata* against the toxins produced by *Corynebacterium diphtheriae*, and observed a stable and strong interaction of bisandrographolide and andrographiside with the protein 1DTP. In their contribution, El-Shiekh et al used the fruits of *Phyllanthus Emblica* to extract luteolin 4′-neohesperidoside (L4N). The final article of this Special Issue evaluated, through in vitro and in vivo assays, whether L4N possesses activity against MRSA, *K. pneumoniae*, fosA-positive Shiga toxin-producing *E. coli* serogroup O111 (STEC O111) and *Bacillus cereus*. Promising results were obtained, particularly against STEC O111 and *K. pneumoniae*.

All the articles published in this Special Issue make a significant contribution to the field of natural products, specifically in terms of antimicrobial properties. Various sources of natural products from different parts of the globe were reported by the authors as a source of bioactive compounds against challenging pathogens, once again highlighting the promising potential of natural products and emphasizing the richness of biodiversity that should be preserved. The scientific contributions achieved with the Special Issue “Antibacterial Agents from Natural Sources” were satisfactory, encouraging a second edition (Special Issue “Antibacterial Agents from Natural Source, 2nd Edition”), which is currently open for submissions.

## References

[1] Newman D.J., Cragg G.M. (2020). Natural Products as Sources of New Drugs over the Nearly Four Decades from 01/1981 to 09/2019. J. Nat. Prod..

